# Robotic Surgical System Adoption and Radical Prostatectomy Volume in Japan: A Nationwide Hospital-Level Longitudinal Difference-in-Differences Study

**DOI:** 10.34172/ijhpm.9571

**Published:** 2026-08-22

**Authors:** Satoshi Funada, Jun Kamei, Yan Luo, Rei Goto

**Affiliations:** ^1^Health Technology Assessment Unit, Department of Preventive Medicine and Public Health, Keio University School of Medicine, Tokyo, Japan.; ^2^Department of Urology, Graduate School of Medicine, The University of Tokyo, Tokyo, Japan.; ^3^Department of Next-Generation Organ Transplantation, Graduate School of Medicine, The University of Tokyo, Tokyo, Japan.; ^4^Graduate School of Business Administration, Keio University, Yokohama, Japan.; ^5^Graduate School of Health Management, Keio University, Fujisawa, Japan.

**Keywords:** Prostate Cancer, Radical Prostatectomy, Robotic Surgery

## Abstract

**Background:**

Robotic-assisted radical prostatectomy (RARP) has become the dominant approach for prostate cancer surgery, replacing open and laparoscopic procedures. However, whether the introduction of robotic surgical systems has increased the overall number of radical prostatectomies (RPs) remains unclear, particularly within social health insurance systems.

**Methods:**

We conducted a nationwide longitudinal observational study using hospital-level data from Japan between 2016 and 2023. Hospitals performing at least one RP annually and reporting complete data on robotic surgical system status and RP volume across the study period were included in the primary analysis. The primary outcome was the annual number of RPs per hospital. Using a staggered difference-in-differences (DID) design, we compared hospitals that adopted robotic surgical systems with hospitals that had not yet adopted them and estimated the association between adoption and annual hospital-level RP volume.

**Results:**

A total of 288 hospitals were categorized as 189 previous adopters, 77 new adopters, and 22 non-adopters. New adopters demonstrated a sustained increase in RP volume, rising from 2608 procedures in 2016 to 4371 in 2023, whereas RP volumes among previous adopters and non-adopters remained stable. Compared with hospitals that had not yet adopted robotic surgical systems, adoption was associated with an increase of 17.4 RPs per hospital per year (95% CI, 10.7 to 24.1). No significant differences were observed between groups during the pre-adoption period, consistent with the parallel trends assumption.

**Conclusion:**

In Japan’s social health insurance context, adoption of robotic surgical systems was associated with a significant and sustained rise in RP volume. These findings highlight the need for further research to determine whether the observed increase reflects changes in patient selection, referral patterns, clinical appropriateness, or other mechanisms, as well as the associated clinical and economic implications.

## Background

**Key Messages**

**Implications for policy makers**

- Adoption of robotic surgical systems was associated with a substantial increase in radical prostatectomy (RP) volume.
- Several mechanisms may explain this increase, including changes in patient selection, referral patterns, treatment practices, or patient preferences. However, the available hospital-level data did not allow us to determine the relative contribution of these factors.
- Policy-makers and clinicians may consider establishing monitoring and evaluation frameworks to assess patient selection, clinical outcomes, and economic impact, thereby promoting the appropriate, equitable, and cost-effective use of robotic surgery.

**Implications for the public**

This national study examined how the introduction of robotic surgical systems has influenced the number of prostate cancer surgeries performed in Japan. The findings suggest that adoption of robotic surgical systems was associated with an increase in radical prostatectomy (RP) volume at adopting hospitals. Several factors may contribute to this increase, including changes in patient selection, referral patterns, treatment practices, or patient preferences. However, the available hospital-level data did not allow us to determine the underlying reasons for the observed increase. While robotic surgery may offer benefits such as less invasive treatment and faster recovery for some patients, ongoing evaluation of patient selection, clinical outcomes, and healthcare costs is important to ensure that this technology is used appropriately, effectively, and equitably.

Robotic-assisted radical prostatectomy (RARP), with its three-dimensional visualization and precise instrument control, has been regarded as a “Game-Changer” in radical prostatectomy (RP), enabling anatomically refined techniques to improve oncologic and functional outcomes.^1-3^ Since its introduction, the proportion of RARP among RP procedures has steadily increased, while open RP and laparoscopic RP have declined.^4-8^ However, it remains unclear whether the overall number of RPs has increased after robotic surgical system adoption. A rise in RP volume could reflect several mechanisms, including changes in patient selection, referral patterns, or treatment practices.^9^ However, if RP volume increases beyond evidence-based indications, concerns regarding overtreatment, treatment-related complications such as urinary incontinence and erectile dysfunction, and higher healthcare costs may arise.^10^ Determining whether robotic surgical system adoption increases RP volume is thus clinically and economically relevant.

 A methodological challenge is distinguishing increases in RP volume due to robotic surgical system adoption from underlying incidence trends. To address this, institutions can be classified by adoption status, and RP volumes compared before and after robotic surgical system adoption. This difference-in-differences (DID) framework allows evaluation of whether newly adopting institutions show increases beyond temporal trends in other groups. This approach was recently applied in the United States, where a DID study demonstrated that robotic surgical system adoption significantly increased RP volume.^11^ However, comparable evidence from countries with different healthcare delivery systems and regulatory frameworks remains limited.

 In Japan, robotic-assisted surgical systems were approved in 2009, and RARP was covered by social health insurance starting in 2012.^12^ Since then, RARP has rapidly diffused nationwide, and Japan has become one of the largest adopters of robotic surgical systems after the United States.^12^ This unique context provides an opportunity to examine the relationship between robotic surgical system adoption and RP volumes within a social health insurance system, where economic incentives, patient selection, and diffusion patterns may differ from those observed in the United States. Examining this relationship in Japan can help strengthen the evidence base and provide policy-relevant insights for other countries regarding RP volume changes associated with the introduction of robotic surgery.

 This nationwide study leverages the institutional context of Japan to examine whether robotic surgical system adoption is associated with changes in hospital-level RP volume. We classified institutions according to their robotic surgical system adoption status and compared RP volume trends during the observation period. We then used a DID approach to estimate the association between robotic surgical system adoption and changes in RP volume by comparing newly adopting institutions with non-adopting institutions.

## Methods

###  Study Design

 This longitudinal observational study used hospital-level data in Japan to evaluate the association between the adoption of robotic surgical systems and the volume of RP performed using a DID analytical framework. The study followed the Strengthening the Reporting of Observational Studies in Epidemiology (STROBE) reporting guidelines.^13^

###  Setting

 The study was conducted in Japan and included hospital data from fiscal year (FY) 2016 through FY2023. The primary exposure was the adoption of a robotic surgical system, and the primary outcome was the annual volume of RPs. Data collection and exposure classification were based on yearly updates of publicly available administrative data sources.

###  Data Source

 Data were compiled by Wellness Co., Ltd.^14^ from three public data sources:

1. Regional Bureaus of Health and Welfare^15^: Provided hospital characteristics, including number of beds, physician workforce, prefecture, and ownership type, as published in official surveys and reports.
2. The survey on the effects of the introduction of Diagnosis Procedure Combination (DPC)^16^: The DPC is a nationwide patient classification system launched in 2003 to support per-diem reimbursement for acute care under the DPC-based Per-Diem Payment System (DPC/PDPS).^17^ The Ministry of Health, Labour and Welfare (MHLW) supplied annual data on surgeries and average lengths of stay. The survey included both DPC/PDPS hospitals and non-DPC/PDPS hospitals reimbursed fee-for-service, covering over half of all hospitals in Japan. This dataset captured nearly 90% of RPs reported in the National Database, a mandatory nationwide claims database.^18^
3. Administrative reports on hospital bed function^19^: Initiated in 2014 under the Medical Care Act,^20^ these reports support regional healthcare planning. All hospitals (excluding psychiatric) and inpatient clinics must submit annual reports to prefectural governments as of July 1. Published by the MHLW, they provide ward-level information (bed numbers, functional classification) and facility-level details, including robotic surgical system installation.

The timing of data collection differed across data sources. Hospital characteristics reflected information available as of January 1 of each FY. RP volume from the survey on the effects of the introduction of DPC represented procedures performed during the entire FY (April 1 to March 31). Robotic surgical system status from the Administrative Reports on Hospital Bed Function reflected facility status as of July 1 of each FY. The three datasets were linked annually at the hospital level using hospital names as the primary identifier. When discrepancies in hospital names were identified across datasets, linkage was verified using additional information, including corporate identification numbers, hospital addresses, and ownership information. Records with apparent inconsistencies across data sources were manually reviewed and reconciled whenever possible. These data sources were linked at the hospital level to construct a longitudinal panel dataset spanning FY2016 to FY2023.

###  Study Population

 The study population included hospitals listed in the survey on the effects of the introduction of DPC that reported performing at least one RP for prostate cancer during the study period. Hospitals without RP procedures during the study period were excluded. The primary analysis was restricted to hospitals with complete data on robotic surgical system status and RP volume across all FYs from FY2016 to FY2023. The hospital selection process is summarized in Figure 1. A total of 3501 hospitals were included in the DPC database in 2016, increasing to 6304 in 2023. Hospitals that performed at least one RP procedure and had available information on robotic surgical system status in a given FY constituted the all-case cohort used in the sensitivity analysis. For the primary analysis, hospitals were further restricted to those with complete information on robotic surgical system status and RP volume for every FY from 2016 through 2023. The final complete-case cohort comprised 288 hospitals, including 189 previous adopters, 77 new adopters, and 22 non-adopters.

**Figure 1:**
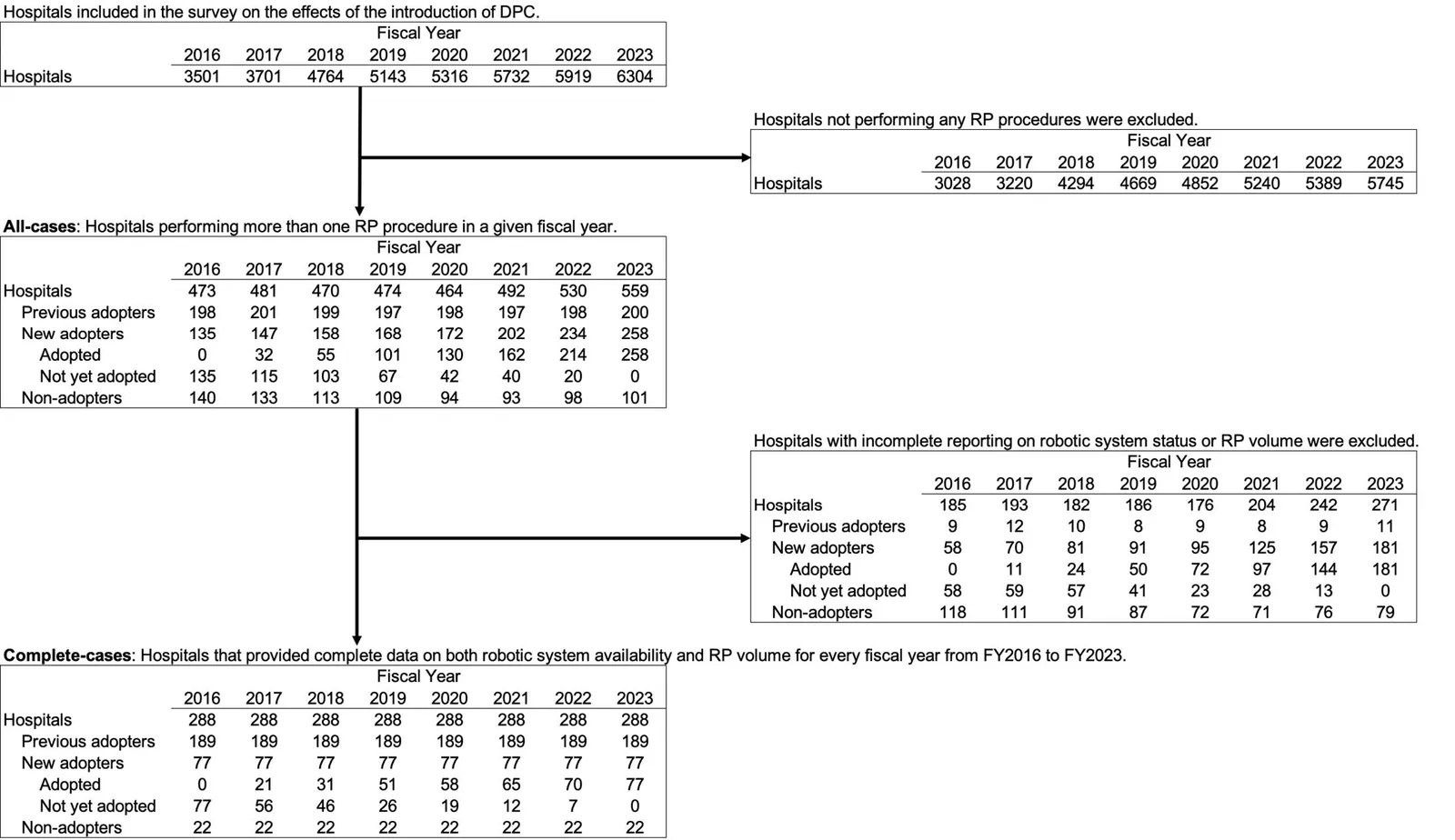
Flow Diagram of Hospital Selection for the Complete-Case and All-Case Analyses. All hospitals were initially selected from the survey on the effects of the introduction of DPC. Hospitals that did not perform any RP during the study period were excluded. The remaining hospitals that performed at least one RP procedure and had non-missing information on both robotic surgical system status and RP volume in a given FY constituted the all-case cohort used in the sensitivity analysis. For the primary complete-case analysis, hospitals were further restricted to those with complete information on both robotic surgical system availability and RP volume for every FY from FY2016 through FY2023. Abbreviations: DPC, Diagnosis Procedure Combination; FY, fiscal year; RP, radical prostatectomy.

###  Study Variables

####  Outcome

 The primary outcome was the annual number of RPs performed at each hospital, as reported in the survey on the effects of the introduction of DPC. RP procedures were identified from DPC category 110080xx01xxxx, corresponding to prostate cancer and related diagnoses. The outcome included all RP procedures regardless of surgical approach, including RARP (K843-4), minimum-incision endoscopic RP (K843-3), laparoscopic RP (K843-2), and open RP (K843). Annual RP volume was measured for each FY (April 1 to March 31).

####  Exposure

 Exposure was defined as the adoption of a robotic surgical system, based on data from the Administrative Reports on Hospital Bed Function. This dataset records the number of adopted robotic surgical systems at each hospital as of July 1 of each FY. Hospitals were classified as having newly adopted a robotic surgical system in the first FY in which one or more robotic surgical systems were reported after no robotic surgical systems had been reported in the previous FY. Based on the timing of adoption, hospitals were categorized as follows:

- Previous adopters: Already had one or more robotic surgical systems installed by FY2016
- New adopters: Newly adopted robotic surgical systems between FY2017 and FY2023
- Non-adopters: Had not adopted by FY2023

####  Hospital Characteristics

 The following hospital characteristics were included in the analysis: number of beds, total number of physicians, annual number of inpatient admissions, total number of surgeries performed annually, geographic region (prefecture), and ownership type (eg, national, public, medical corporation). Prefecture size was categorized into three groups based on the total population: large (≥3 million population), medium (1 to 3 million), and small (<1 million). Population data were obtained from the latest national census.^21^

###  Statistical Analysis

####  Descriptive Analysis

 Descriptive statistics for hospital characteristics at baseline (FY2016) were summarized by robotic surgical system adoption status (previous adopters, new adopters, and non-adopters). For each group, we calculated the total number of RPs performed from FY2016 to FY2023, the mean annual number of RPs per hospital with standard deviation (mean [SD]) and reported trends in robotic surgical system adoption and RP volume over time.

####  Primary Analysis

 The primary analysis was conducted using a complete-case cohort consisting of hospitals with complete information on robotic surgical system status and RP volume for every FY from FY2016 through FY2023 (Figure 1). Because hospitals adopted robotic surgical systems at different times, traditional DID methods that assume a single common treatment period may lead to biased estimates due to dynamic treatment effects.^22^ To address this, we applied a staggered DID design to estimate the association between robotic surgical system adoption and changes in RP volume, comparing new adopters with non-adopters. Hospitals classified as previous adopters were included in the analytic dataset and descriptive analyses because they provide important context regarding the nationwide diffusion of robotic surgical systems. However, they did not contribute to the identification of treatment effects because robotic surgical system adoption had occurred before the start of the observation period. Hospitals were assigned to adoption cohorts according to the first FY in which one or more robotic surgical systems were reported. Hospitals adopting robotic surgical systems between FY2017 and FY2023 were assigned to the corresponding adoption-year cohort. We employed the method developed by Callaway and Sant’Anna to estimate group-time average treatment effect (ATT) for each adoption cohort, defined by the FY in which robotic surgery was introduced.^22^ These cohort-specific estimates were aggregated to calculate the overall ATT, which can be interpreted as the difference in RP volume for adopting hospitals relative to hospitals that had not yet adopted robotic surgical systems. In this framework, hospitals that had not yet adopted robotic surgical systems served as controls until their adoption year, whereas hospitals that never adopted robotic surgical systems remained in the control group throughout the study period. Group-time treatment effects were estimated using the doubly robust estimator, which combines outcome regression and inverse probability weighting, with standard errors clustered at the hospital level. Additionally, an event-study analysis was conducted to assess dynamic treatment effects over time, with the year of adoption defined as event time zero. Negative event times represent years before adoption, whereas positive event times represent years after adoption.

####  Sensitivity and Further Analyses

 We conducted several sensitivity and additional analyses to assess the robustness of our findings. First, we performed an all-case analysis using all available hospital-year observations with non-missing robotic surgical system status and RP volume, regardless of whether hospitals had complete data for every FY from FY2016 through FY2023 (Figure 1). This analysis was conducted to evaluate the potential impact of the complete-case restriction on the study findings. Second, as an alternative estimation strategy, we applied the stacked DID approach developed by Gardner.^23^ For each adoption cohort, we stacked the data relative to the adoption year and estimated event-time-specific treatment effects using a two-stage regression framework. Hospital and year fixed effects were partialed out in the first stage, and treatment effects were estimated in the second stage. Standard errors were clustered at the hospital level. Because the stacked DID approach relies on a different identification strategy from the Callaway and Sant’Anna framework, it was used as a complementary sensitivity analysis to assess the robustness of the findings across multiple adoption timings. All analyses were conducted between October 2024 and August 2025 using R software (version 4.4.1; R Foundation for Statistical Computing, Vienna, Austria). DID models were implemented using the “did”^24^ and “did2s”^25^ packages. The R code used for data processing, cohort assignment, and statistical analyses is available at Supplementary file 1.

## Results

###  Baseline Characteristics

Table 1 summarizes baseline hospital characteristics by adoption status. Previous adopters had more beds, physicians, annual new inpatients, and surgeries than new adopters and non-adopters. They were also more often located in large prefectures and operated by national or public organizations. Compared with non-adopters, new adopters consistently had greater capacity and surgical volume. On average, new adopters had 576 beds and 175 physicians, versus 479 beds and 117 physicians among non-adopters. They also admitted more new inpatients (16 546 vs. 11 945) and performed more surgeries (519 vs. 413). Non-adopters were more often located in medium-sized prefectures and more frequently operated by public or private institutions rather than national facilities. Characteristics of hospitals included in the primary analysis were generally comparable to those excluded because of incomplete data, although excluded hospitals tended to be somewhat smaller (Table S1, Supplementary file 2).

**Table 1:** Baseline Characteristics of Included Hospitals

**Variable**	**Previous Adopters**	**New Adopters**	**Non-adopters**
	**N = 189**	**N = 77**	**N = 22**
Number of beds, mean (SD)	667 (245)	576 (184)	479 (199)
Number of physicians^a^, mean (SD)	246 (167)	175 (122)	117 (124)
Annual new inpatient cases, mean (SD)	18 362 (7386)	16 546 (5984)	11 945 (5147)
Total number of surgeries, mean (SD)	604 (253)	519 (195)	413 (264)
Prefecture size, n (%)			
Large (≥3 million population)	109 (57.7%)	53 (68.8%)	8 (36.4%)
Medium (1 to 3 million population)	62 (32.8%)	19 (24.7%)	13 (59.1%)
Small (<1 million population)	18 (9.5%)	5 (6.5%)	1 (4.5%)
Ownership, n (%)			
National government	53 (28.0%)	6 (7.8%)	6 (27.3%)
Public organizations	67 (35.4%)	46 (59.7%)	8 (36.4%)
Non-profit medical corporation	22 (11.6%)	4 (5.2%)	1 (4.5%)
Private medical schools	27 (14.3%)	13 (16.9%)	5 (22.7%)
Other	20 (10.6%)	8 (10.4%)	2 (9.1%)

Abbreviations: FY, fiscal year; SD, standard deviation. ^a^ Physician data were unavailable in FY2016 and were instead obtained from FY2017.

###  Trends of Radical Prostatectomy

Figure 2 and Table S2 present trends in the mean annual number of RPs per hospital according to robotic surgical system adoption status from FY2016 to FY2023. During this period, the number of new adopters steadily increased from 0 in FY2016 to 77 in FY2023 (0, 21, 31, 51, 58, 65, 70, and 77, respectively). Throughout the study period, previous adopters consistently performed a higher number of RPs per hospital compared with new adopters and non-adopters. While the number among both previous adopters and non-adopters remained relatively stable, new adopters exhibited a gradual increase in surgical volume, particularly from FY2020 onward. The mean number of RPs per hospital in FY2016 was comparable between new adopters and non-adopters (33.9 and 34.4, respectively), but by FY2023, new adopters had increased to 56.8, whereas non-adopters reached only 39.3.

**Figure 2:**
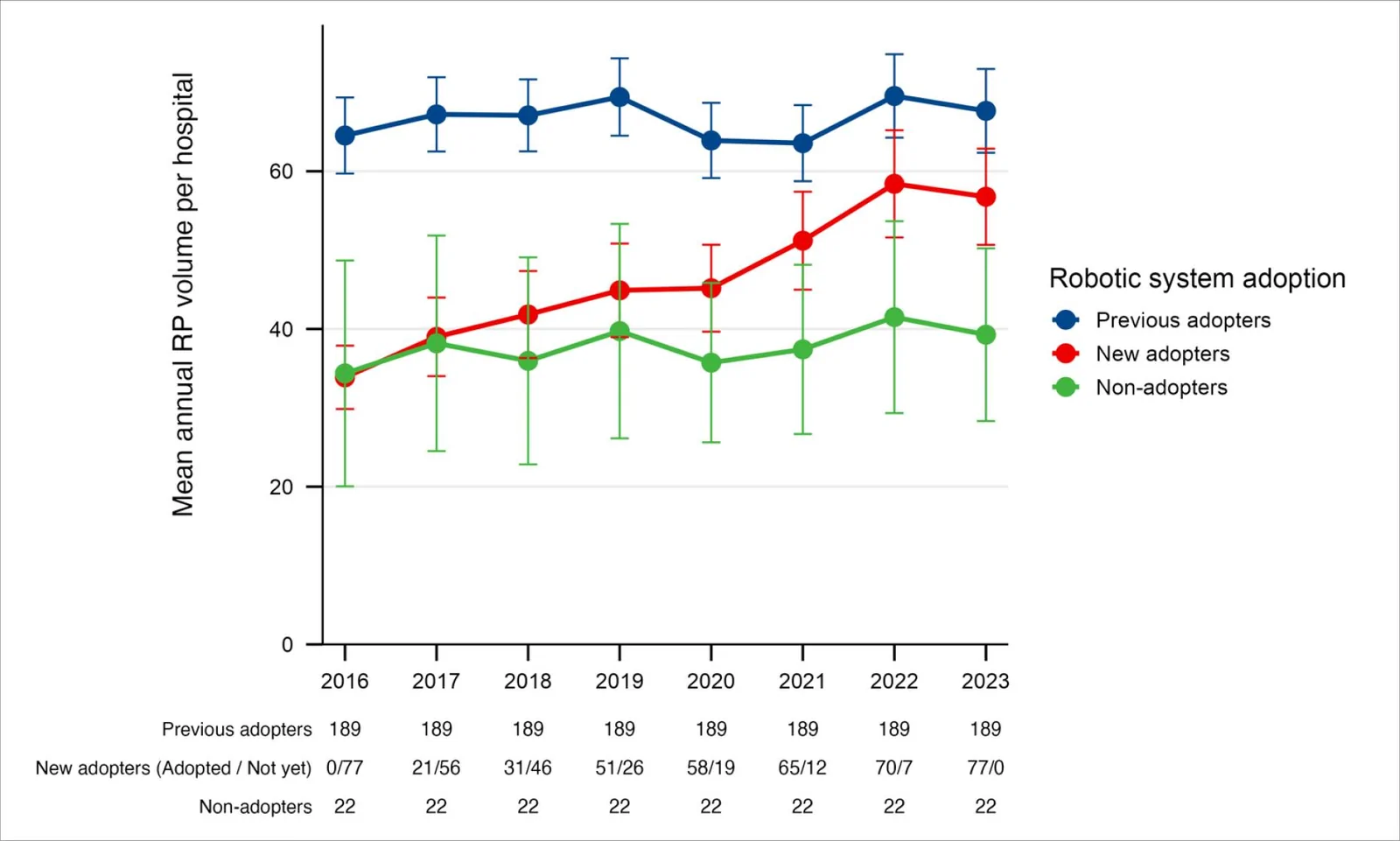
Trends in the Mean Annual Number of Radical Prostatectomies per Hospital by Robotic Surgical System Adoption Status, FY2016–FY2023. Values represent mean annual RPs per hospital. Error bars indicate 95% confidence intervals. For new adopters, “Adopted/Not yet” indicates the number of hospitals that had already adopted robotic surgical systems and those that had not yet adopted in each FY. Abbreviations: FY, fiscal year; RP, Radical Prostatectomy.

###  Primary Results

Figure 3 and Table 2 show the results of the DID analysis estimating the impact of robotic surgical system adoption on the number of RPs performed at the hospital level, comparing newly adopting institutions with non-adopting institutions. The event-study specification revealed no statistically significant differences in RP volume between adopting and non-adopting hospitals in the pre-adoption period (years –6 to –1), providing support for the parallel trends assumption. Starting from the year of adoption (year 0), RP volume increased relative to non-adopting hospitals. The estimated ATT at year 0 was 14.26 (95% confidence interval [CI, 10.07 to 18.45) and remained significantly positive throughout most of the post-adoption period. Estimates ranged from 11.66 to 20.30 additional RPs per hospital, indicating a sustained increase in RP volume following robotic surgical system adoption. Cohort-specific treatment effects according to FY of adoption are presented in Table 2. The overall ATT was 17.40 (95% CI, 10.71 to 24.10), indicating a significant and sustained increase in RP volume following robotic surgical system adoption.

**Figure 3:**
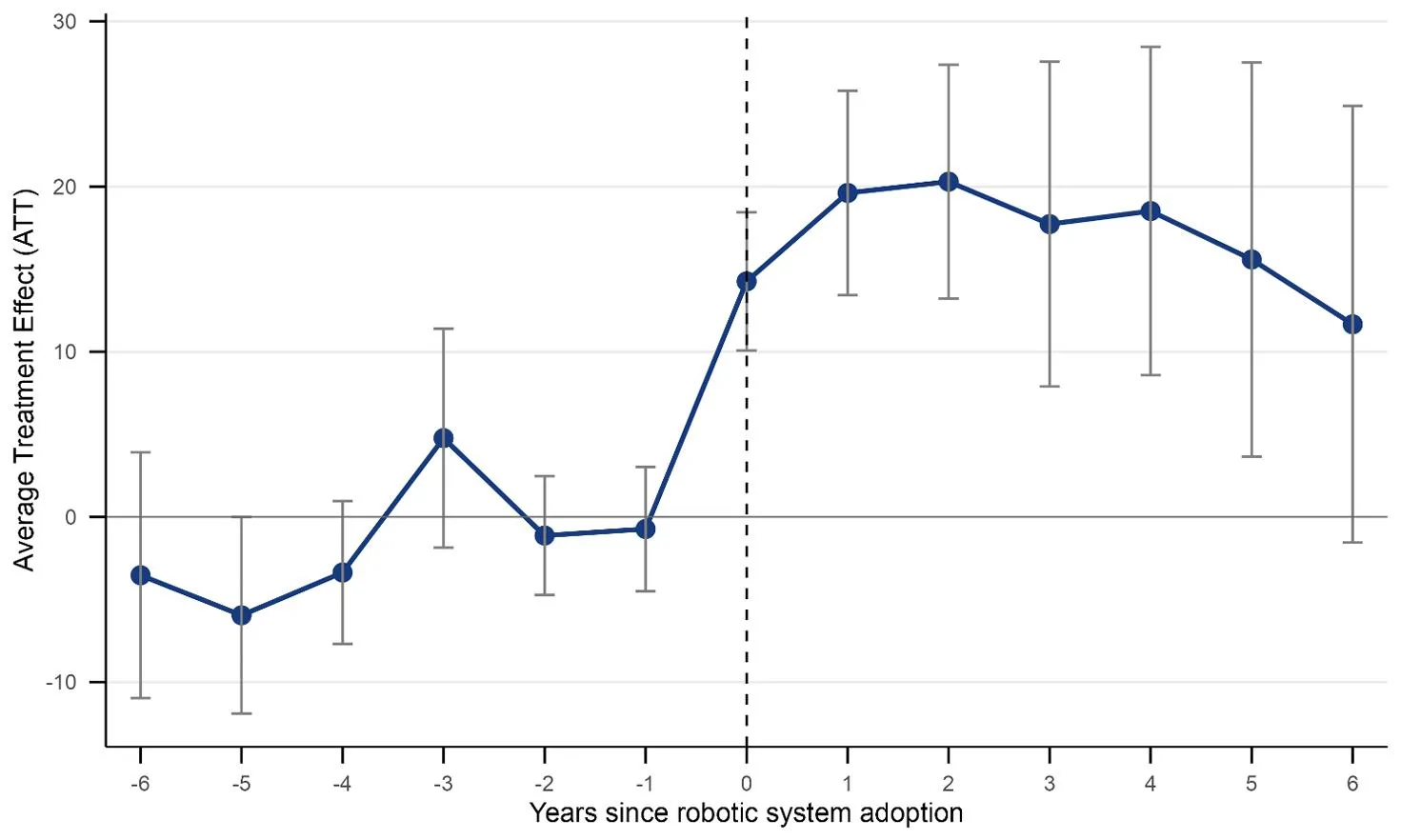
Estimated Average Treatment Effects of Robotic Surgical System Adoption on Radical Prostatectomy. Event-study estimates using a staggered difference-in-differences approach. The dashed vertical line indicates the fiscal year of robotic surgical system adoption (event time 0). Negative event times represent years before adoption, and positive event times represent years after adoption. Error bars indicate 95% confidence intervals. Abbreviation: ATT, average treatment effect.

**Table 2:** Estimated Average Treatment Effects on Radical Prostatectomy

	**No. of Hospitals**	**Average Treatment Effect**	**95% CI (Lower)**	**95% CI (Upper)**
**Years since adoption**				
-6		-3.53	-10.97	3.91
-5		-5.96	-11.91	-0.01
-4		-3.37	-7.69	0.96
-3		4.77	-1.86	11.39
-2		-1.13	-4.73	2.47
-1		-0.73	-4.49	3.02
0		14.26	10.07	18.45
1		19.62	13.43	25.80
2		20.30	13.22	27.38
3		17.73	7.90	27.57
4		18.52	8.58	28.46
5		15.59	3.65	27.52
6		11.66	-1.55	24.88
**Groups by year of adoption**				
FY2017	21	13.68	3.88	23.49
FY2018	10	14.23	6.35	22.11
FY2019	20	20.34	8.28	32.40
FY2020	7	24.96	8.98	40.95
FY2021	7	27.62	12.88	42.36
FY2022	5	22.92	5.59	40.26
FY2023	7	12.08	1.37	22.80
**Overall average**		17.40	10.71	24.10

Abbreviations: CI, confidence interval; FY, fiscal year. Event time was defined relative to the FY of robotic surgical system adoption (event time 0). Negative values indicate years before adoption and positive values indicate years after adoption.

###  Sensitivity Analysis

 To assess the robustness of our findings, we conducted a sensitivity analysis including all hospitals that reported at least one RP procedure during the study period, regardless of whether they reported in every FY (“all-cases” in Figure 1). Trends in RP volume by robotic surgical system adoption group from FY2016 to FY2023 were consistent with the primary analysis (Figure S1 and Table S3). Trends were broadly consistent with those observed in the primary complete-case analysis. Event-study estimates using the staggered DID approach also showed a substantial post-adoption increase in RP volume, with no evidence of differential pre-trends (Figure S2). Treatment effects remained positive throughout most of the post-adoption period (Table S4). Group-specific ATTs by adoption year showed similar patterns, with an overall ATT of 11.95 (95% CI, 8.48 to 15.41).

 We additionally applied a stacked DID approach as a sensitivity analysis, aligning event time relative to each hospital’s year of robotic surgical system adoption. As shown in Figure S3 and Table S5, robotic surgical system adoption was associated with positive post-adoption treatment effects, ranging from 3.66 to 10.69 additional RPs per hospital. Although the magnitude of the effects was smaller than those observed in the staggered DID analysis, the overall pattern remained consistent (Figure S4).

## Discussion

###  Principal Findings

 Using nationwide open data from Japan, we analyzed temporal trends in RP following the adoption of robotic surgical systems. Compared with both previously adopting and non-adopting hospitals, newly adopting hospitals exhibited greater increases in RP volume over time. In the staggered DID analysis comparing new adopters with non-adopters, no meaningful differences were observed during the pre-adoption period, providing support for the parallel trends assumption. In contrast, the post-adoption period showed a significant increase in RP volume. Sensitivity analyses, including those incorporating hospitals with missing FYs and a stacked DID approach, confirmed the robustness of these findings. Although the estimated effect sizes differed between the staggered DID and stacked DID analyses, both approaches demonstrated a positive association between robotic surgical system adoption and RP volume. Given that these methods rely on different identification strategies, the consistency in the direction of the estimates supports the robustness of the findings.

###  Interpretation of Findings

 In this nationwide analysis, robotic surgical system adoption was associated with a persistent increase in RP volume during most of the post-adoption period. Although the mechanisms underlying this association could not be determined from the available data, possible explanations include patient redistribution between hospitals, changes in referral patterns, expansion of surgical indications, or market expansion.^11^ If the observed increase reflects expansion of surgical indications beyond evidence-based practice, concerns regarding overtreatment, complications, and healthcare costs may arise. Moreover, advanced technologies may increase physician–patient information asymmetry and could potentially contribute to supplier-induced demand, in which patients consent without fully understanding risks and benefits. Conversely, if the increase reflects market expansion, this may benefit some patients by enabling surgery for individuals who were previously considered unsuitable candidates because of disease complexity or technical limitations. Because this study was based on hospital-level data, it was not possible to determine which of these mechanisms contributed to the observed increase in RP volume. These findings highlight the importance of evaluating patient characteristics and clinical outcomes following robotic adoption to determine whether increased procedure volume reflects appropriate care.

###  Comparison With Other Studies

 A study from the United States using a DID approach also reported an increase in RP volume following robotic surgical system adoption.^11^ Similar to the present analysis, it applied a facility-level staggered DID design and found a significant post-adoption rise. The concordance of findings across countries with different healthcare systems suggests that the association may be generalizable. Importantly, that study also conducted a market-level DID analysis and found that part of the increase reflected patient transfers (“business stealing”). Prior evidence from Japan has suggested that business stealing may occur following the introduction of robotic surgical systems, as hospitals without robotic surgical systems experienced longer intervals between RPs when a robotic-capable hospital appeared nearby.^26^ Although RP volume increased after robotic surgical system adoption in the present study, the available data did not permit direct evaluation of patient redistribution across hospitals or other market-level mechanisms. Therefore, it was not possible to determine whether the observed increase reflected business stealing, market expansion, or a combination of both. A more definitive assessment would require direct evaluation of market-level effects and historical adoption data for earlier adopters, which were unavailable in the present study.

###  Implications of Findings

 In a social health insurance setting, this nationwide DID analysis showed that robotic surgical system adoption was associated with increased RP volume. Together with previous findings from the United States, these results provide internationally relevant evidence for countries considering adoption, particularly those with universal coverage. While the observed increase in RP volume may reflect several mechanisms, the underlying causes remain uncertain and warrant further investigation using patient-level data. Evaluations should extend beyond surgical volume to examine patient characteristics before and after robotic surgical system adoption, assess clinical outcomes such as complication rates, and determine the impact on healthcare expenditures. Moreover, assessments should include not only new adopters but also previous adopters and non-adopters, enabling a comprehensive understanding of system-wide effects. This broader perspective would allow policy-makers to move beyond a narrow focus on the technical merits of robotic surgery and consider its wider clinical, economic, and policy implications when making decisions about adoption and dissemination.

###  Strengths and Limitations of the Study

 This study has several strengths. First, it utilized a comprehensive nationwide dataset covering nearly 90% of all RPs reported in Japan, thereby ensuring high representativeness. Second, the methodological approach was robust, incorporating a staggered DID design, multiple sensitivity analyses, and a stacked DID approach to validate the consistency of the findings. Third, the consistency of findings between Japan and the United States, despite substantial differences in healthcare financing and delivery, supports the robustness of the observed association across different healthcare settings.

 Nevertheless, certain limitations should be acknowledged. First, although the dataset was extensive, it was not exhaustive, and some hospitals had missing data for certain FYs. In addition, the number of hospitals that never adopted robotic surgical systems during the study period was relatively small, which may limit the precision of comparisons between adopting and non-adopting hospitals. However, the results of the primary complete-case analysis were generally consistent with those of the all-case sensitivity analysis, supporting the robustness of the main findings. Second, robotic surgical system status was measured as of July 1 of each FY, whereas RP volume was measured for the entire FY (April 1 to March 31). As a result, the adoption year may have included both pre-adoption and post-adoption periods, potentially leading to exposure misclassification. Third, certain hospital- or region-level factors, including regional population size, were not included in the final DID models because of model instability and convergence issues. As a result, the possibility of residual confounding should be considered when interpreting the findings. Fourth, cohort-specific treatment effects showed some variation across adoption cohorts. However, the study was not designed to formally investigate sources of heterogeneity according to adoption timing, and several cohort-specific estimates were associated with wide CIs. Therefore, differences among cohort-specific estimates should be interpreted cautiously. Fifth, the analysis was based solely on hospital-level data, limiting the ability to determine the mechanisms underlying the observed increase in RP volume. Accordingly, the findings should be interpreted as associations with hospital-level RP volume rather than evidence regarding the mechanisms responsible for volume changes. Access to patient-level data, including age, pathological characteristics, disease risk, and treatment selection, would help clarify whether the observed increase reflects changes in patient case-mix, referral patterns, treatment indications, or other factors. Further investigation using such detailed data is warranted.

## Conclusions

 In Japan’s social health insurance system, adoption of robotic surgical systems was associated with a sustained increase in RP volume at the hospital level. These findings, consistent with evidence from the United States, may help countries with similar health systems anticipate potential procedural impacts following robotic surgical system adoption. However, the underlying mechanisms responsible for the observed increase could not be determined from the available hospital-level data and warrant further investigation using patient-level information. Future evaluations should assess patient characteristics, clinical outcomes, and economic consequences to ensure that robotic surgical systems are adopted and used appropriately.

## Disclosure of artificial intelligence (AI) use

 The authors used ChatGPT (OpenAI, San Francisco, CA, USA) to assist with English editing and to improve clarity and style. All analyses and interpretations were conducted and verified by the authors, who take full responsibility for the final manuscript.

## Ethical issues

 This study used publicly available hospital-level data and was therefore exempt from institutional review board (IRB) approval and the requirement for informed consent, in accordance with the Ethical Guidelines for Medical and Health Research Involving Human Subjects issued by the MHLW of Japan.

## Conflicts of interest

 Jun Kamei reports lecture fees from Kowa, Kyorin, Bayer, Sanofi, Kissei, and Astellas, outside the submitted work.

## Data availability statement

 The data used in this study were derived from publicly available sources published by the MHLW of Japan and related governmental agencies, including the Survey on the Effects of the Introduction of DPC, Administrative Reports on Hospital Bed Function, and hospital information published by the Regional Bureaus of Health and Welfare. The R code used for data processing, cohort assignment, and statistical analyses is available at Supplementary file 1.

## Supplementary files


Supplementary file 1. Analysis Code.

Supplementary file 2 contains Tables S1-S5 and Figures S1-S4.
