# Supplementary material for: Robotic Surgical System Adoption and Radical Prostatectomy Volume in Japan: A Nationwide Hospital-Level Longitudinal Difference-in-Differences Study
Source: Int J Health Policy Manag. 2026 Aug 22;15:9571. doi: 10.34172/ijhpm.9571 (PMC13770223; doi:10.34172/ijhpm.9571)
Supplement: Supplementary file 1 [file ijhpm-15-9571-s001.pdf]

Article title: Robotic Surgical System Adoption and Radical Prostatectomy Volume in Japan: A Nationwide Hospital-Level Longitudinal Difference-in-Differences Study

Journal name: International Journal of Health Policy and Management (IJHPM)

Authors' information: Satoshi Funada[1]*, Jun Kamei[2], Yan Luo[3], Rei Goto[4,5]

[1]Health Technology Assessment Unit, Department of Preventive Medicine and Public Health, Keio University School of Medicine, Tokyo, Japan.

[2]Department of Urology, Graduate School of Medicine, The University of Tokyo, Tokyo, Japan.

[3]Department of Next-Generation Organ Transplantation, Graduate School of Medicine, The University of Tokyo, Tokyo, Japan.

[4]Graduate School of Business Administration, Keio University, Yokohama, Japan.

[5]Graduate School of Health Management, Keio University, Fujisawa, Japan.

***Correspondence to:** Satoshi Funada; Email: sfunada@keio.jp

Citation: Funada S, Kamei J, Luo Y, Goto R. Robotic surgical system adoption and radical prostatectomy volume in Japan: a nationwide hospital-level longitudinal difference-in-differences study. Int J Health Policy Manag. 2026;15:9571. doi:10.34172/ijhpm.9571

Supplementary file 1. Analysis Code

```
# ============================================================
# Robotic Surgical System Adoption and Radical Prostatectomy Volume
# Analysis Code
# Complete-case analysis, all-case sensitivity analysis,
# staggered DID, and stacked DID
# ============================================================

rm(list = ls())

# ============================================================
# README
# ============================================================
# This script reproduces the analytic workflow used to estimate
# the association between robotic surgical system adoption and
# hospital-level radical prostatectomy (RP) volume.
#
# The raw linked hospital-level dataset is not redistributed.
# To run this script, users should prepare a hospital-year panel
# dataset with one row per hospital-year.
#
# Required input columns:
#     hospital_id              Unique numeric hospital identifier
#     fiscal_year              Fiscal year
#     rp_volume                Annual number of radical prostatectomies
#     rp_los                   Average length of stay for radical prostatectomy
#     robot_system_count       Number of robotic surgical systems reported
#
# Expected structure:
#     hospital_id | fiscal_year | rp_volume | rp_los | robot_system_count
#
# Notes:
#     - Missing values are treated as missing information, not as zero.
```

```
#     - The adoption year is defined before restricting hospitals by RP volume.
#     - Complete-case analysis includes hospitals with complete RP volume,
#         RP length of stay, and robotic system status across all fiscal years.
#     - All-case sensitivity analysis follows the original analytic definition:
#             1) hospitals with robotic system information in at least one fiscal year;
#             2) hospital-year observations with RP volume >= 1 and RP length of stay >= 1;
#             3) missing fiscal years are allowed.
#     - Previous adopters are retained for descriptive classification but
#         excluded from DID estimation because robotic system adoption occurred
#         before the observation period.
#     - For stacked DID, hospital_id is treated as the original numeric identifier
#         to reproduce the original model specification.

# ============================================================
# 0. Packages
# ============================================================

required_packages <- c(
   "dplyr",
   "tidyr",
   "purrr",
   "stringr",
   "readr",
   "did",
   "did2s",
   "broom",
   "fixest"
)

missing_packages <- required_packages[
   !required_packages %in% rownames(installed.packages())
]

if (length(missing_packages) > 0) {
   stop(
      "The following packages are not installed: ",
      paste(missing_packages, collapse = ", ")
   )
}

invisible(lapply(required_packages, library, character.only = TRUE))

# ============================================================
# 1. Settings
# ============================================================

input_file <- "data/hospital_year_panel.csv"

target_years <- 2016:2023
first_year <- min(target_years)
last_year    <- max(target_years)

set.seed(12345)

# ============================================================
# 2. Load and check input data
# ============================================================

data_raw <- read_csv(input_file, show_col_types = FALSE)

required_cols <- c(
   "hospital_id",
   "fiscal_year",
   "rp_volume",
   "rp_los",
```

```
  "robot_system_count"
)

missing_cols <- setdiff(required_cols, names(data_raw))

if (length(missing_cols) > 0) {
  stop(
    "The following required columns are missing: ",
    paste(missing_cols, collapse = ", ")
  )
}

data_raw <- data_raw %>%
  mutate(
    hospital_id = as.numeric(hospital_id),
    fiscal_year = as.integer(fiscal_year),
    rp_volume = as.numeric(rp_volume),
    rp_los = as.numeric(rp_los),
    robot_system_count = as.numeric(robot_system_count)
  ) %>%
  filter(fiscal_year %in% target_years)

# ============================================================
# 3. Define robotic surgical system adoption year
# ============================================================

adoption_year_data <- data_raw %>%
  filter(
    !is.na(robot_system_count),
    robot_system_count > 0
  ) %>%
  group_by(hospital_id) %>%
  summarise(
    adoption_year = min(fiscal_year),
    .groups = "drop"
  )

# ============================================================
# 4. Assign adoption group and DID timing variable
# ============================================================

assign_adoption_group <- function(df, adoption_year_data) {

  df %>%
    left_join(adoption_year_data, by = "hospital_id") %>%
    mutate(
      adoption_group = case_when(
        !is.na(adoption_year) & adoption_year <= first_year ~ "Previous adopters",
        !is.na(adoption_year) & adoption_year > first_year & adoption_year <= last_year ~ "New
adopters",
        is.na(adoption_year) | adoption_year > last_year ~ "Non-adopters",
        TRUE ~ NA_character_
      ),
      G = case_when(
        adoption_group == "New adopters" ~ adoption_year,
        adoption_group == "Non-adopters" ~ 0,
        adoption_group == "Previous adopters" ~ first_year,
        TRUE ~ NA_real_
      ),
      adoption_group = factor(
        adoption_group,
        levels = c("Previous adopters", "New adopters", "Non-adopters")
      )
    ) %>%
    filter(
```

```
      !is.na(rp_volume),
      !is.na(adoption_group),
      !is.na(G)
    )
}

# ============================================================
# 5. Create complete-case and all-case analysis datasets
# ============================================================

create_analysis_data <- function(data_raw, analysis_type = c("complete", "all")) {

  analysis_type <- match.arg(analysis_type)

  if (analysis_type == "complete") {

    # Complete-case hospitals:
    # - observed in every fiscal year
    # - non-missing RP volume in every fiscal year
    # - RP volume >= 1 in every fiscal year
    # - non-missing RP length of stay in every fiscal year
    # - RP length of stay >= 1 in every fiscal year
    # - non-missing robotic surgical system status in every fiscal year
    valid_hospitals <- data_raw %>%
      group_by(hospital_id) %>%
      summarise(
        has_all_years = n_distinct(fiscal_year) == length(target_years),
        complete_robot_status = all(!is.na(robot_system_count)),
        complete_rp_volume = all(!is.na(rp_volume) & rp_volume >= 1),
        complete_rp_los = all(!is.na(rp_los) & rp_los >= 1),
        .groups = "drop"
      ) %>%
      filter(
        has_all_years,
        complete_robot_status,
        complete_rp_volume,
        complete_rp_los
      ) %>%
      pull(hospital_id)

    analysis_data <- data_raw %>%
      filter(
        hospital_id %in% valid_hospitals,
        fiscal_year %in% target_years
      )
  }

  if (analysis_type == "all") {

    # All-case sensitivity analysis:
    # - hospitals must have robotic system information in at least one fiscal year
    # - hospital-year observations must have RP volume >= 1
    # - hospital-year observations must have RP length of stay >= 1
    # - missing fiscal years are allowed
    valid_hospitals <- data_raw %>%
      group_by(hospital_id) %>%
      summarise(
        has_robot_info = any(!is.na(robot_system_count)),
        .groups = "drop"
      ) %>%
      filter(has_robot_info) %>%
      pull(hospital_id)

    analysis_data <- data_raw %>%
      filter(
```

```
            hospital_id %in% valid_hospitals,
            fiscal_year %in% target_years,
            rp_volume >= 1,
            rp_los >= 1
        )
    }

    did_panel_all <- analysis_data %>%
        assign_adoption_group(adoption_year_data)

    did_panel_primary <- did_panel_all %>%
        filter(adoption_group != "Previous adopters")

    list(
        analysis_data = analysis_data,
        did_panel_all = did_panel_all,
        did_panel_primary = did_panel_primary
    )
}

complete_data <- create_analysis_data(data_raw, "complete")
all_case_data <- create_analysis_data(data_raw, "all")

# ============================================================
# 6. Staggered DID using Callaway and Sant'Anna
# ============================================================

get_overall_results <- function(overall_att, analysis_label) {

    if (is.list(overall_att)) {
        overall_att_value <- overall_att$overall.att
        overall_se_value <- overall_att$overall.se
    } else {
        overall_att_value <- as.numeric(overall_att["overall.att"])
        overall_se_value <- as.numeric(overall_att["overall.se"])
    }

    tibble(
        analysis = analysis_label,
        overall_att = overall_att_value,
        se = overall_se_value,
        ci_lower = overall_att_value - 1.96 * overall_se_value,
        ci_upper = overall_att_value + 1.96 * overall_se_value
    )
}

run_staggered_did <- function(did_panel_primary,
                              analysis_label,
                              allow_unbalanced_panel) {

    set.seed(12345)

    att_gt_result <- att_gt(
        yname = "rp_volume",
        tname = "fiscal_year",
        idname = "hospital_id",
        gname = "G",
        data = did_panel_primary,
        control_group = "nevertreated",
        est_method = "dr",
        allow_unbalanced_panel = allow_unbalanced_panel,
        clustervars = "hospital_id",
        bstrap = TRUE,
        biters = 999
    )
```

```
  dynamic_att <- aggte(att_gt_result, type = "dynamic")
  overall_att <- aggte(att_gt_result, type = "simple")
  group_att <- aggte(att_gt_result, type = "group")

  event_study_results <- tibble(
    analysis = analysis_label,
    event_time = dynamic_att$egt,
    att = dynamic_att$att.egt,
    se = dynamic_att$se.egt,
    ci_lower = att - 1.96 * se,
    ci_upper = att + 1.96 * se
  )

  group_att_results <- tibble(
    analysis = analysis_label,
    adoption_year = group_att$egt,
    att = group_att$att.egt,
    se = group_att$se.egt,
    ci_lower = att - 1.96 * se,
    ci_upper = att + 1.96 * se
  )

  overall_att_results <- get_overall_results(overall_att, analysis_label)

  list(
    att_gt_result = att_gt_result,
    dynamic_att = dynamic_att,
    overall_att = overall_att,
    group_att = group_att,
    event_study_results = event_study_results,
    group_att_results = group_att_results,
    overall_att_results = overall_att_results
  )
}

complete_staggered <- run_staggered_did(
  did_panel_primary = complete_data$did_panel_primary,
  analysis_label = "complete",
  allow_unbalanced_panel = FALSE
)

all_case_staggered <- run_staggered_did(
  did_panel_primary = all_case_data$did_panel_primary,
  analysis_label = "all_case",
  allow_unbalanced_panel = TRUE
)

# ============================================================
# 7. Stacked DID
# ============================================================

run_stacked_did <- function(did_panel_primary, analysis_label) {

  adoption_years <- sort(unique(did_panel_primary$G[did_panel_primary$G != 0]))

  stacked_panel <- map_dfr(adoption_years, function(gy) {
    did_panel_primary %>%
      filter(fiscal_year %in% (gy - 1):(gy + 6)) %>%
      mutate(
        event_time = fiscal_year - gy,
        treated = as.integer(fiscal_year >= gy & G == gy),
        stack_group = paste0("G", gy)
      )
  }) %>%
```

```
    filter(event_time %in% c(-1, 0, 1, 2, 3, 4, 5, 6)) %>%
    mutate(
      event_time_factor = relevel(factor(event_time), ref = "-1")
    )

  stacked_model <- did2s(
    data = stacked_panel,
    yname = "rp_volume",
    first_stage = ~ hospital_id + fiscal_year,
    second_stage = ~ i(event_time_factor, treated, ref = "-1"),
    treatment = "treated",
    cluster_var = "hospital_id"
  )

  stacked_event_results <- tidy(stacked_model) %>%
    filter(str_detect(term, "event_time_factor")) %>%
    mutate(
      analysis = analysis_label,
      event_time = as.numeric(str_extract(term, "-?¥¥d+")),
      ci_lower = estimate - 1.96 * std.error,
      ci_upper = estimate + 1.96 * std.error
    ) %>%
    arrange(event_time)

  list(
    stacked_model = stacked_model,
    stacked_panel = stacked_panel,
    stacked_event_results = stacked_event_results
  )
}

complete_stacked <- run_stacked_did(
  did_panel_primary = complete_data$did_panel_primary,
  analysis_label = "complete"
)

all_case_stacked <- run_stacked_did(
  did_panel_primary = all_case_data$did_panel_primary,
  analysis_label = "all_case"
)

# ============================================================
# 8. Summary result objects
# ============================================================

staggered_event_study_results <- bind_rows(
  complete_staggered$event_study_results,
  all_case_staggered$event_study_results
)

staggered_group_att_results <- bind_rows(
  complete_staggered$group_att_results,
  all_case_staggered$group_att_results
)

staggered_overall_att_results <- bind_rows(
  complete_staggered$overall_att_results,
  all_case_staggered$overall_att_results
)

stacked_event_study_results <- bind_rows(
  complete_stacked$stacked_event_results,
  all_case_stacked$stacked_event_results
)
```

```
# Optional descriptive summaries
hospital_counts_by_group <- bind_rows(
  complete_data$did_panel_all %>%
    count(fiscal_year, adoption_group) %>%
    mutate(analysis = "complete"),
  all_case_data$did_panel_all %>%
    count(fiscal_year, adoption_group) %>%
    mutate(analysis = "all_case")
)

rp_volume_trends <- bind_rows(
  complete_data$did_panel_all %>%
    group_by(fiscal_year, adoption_group) %>%
    summarise(
      n_hospitals = n_distinct(hospital_id),
      total_rp_volume = sum(rp_volume, na.rm = TRUE),
      mean_rp_volume = mean(rp_volume, na.rm = TRUE),
      sd_rp_volume = sd(rp_volume, na.rm = TRUE),
      .groups = "drop"
    ) %>%
    mutate(analysis = "complete"),
  all_case_data$did_panel_all %>%
    group_by(fiscal_year, adoption_group) %>%
    summarise(
      n_hospitals = n_distinct(hospital_id),
      total_rp_volume = sum(rp_volume, na.rm = TRUE),
      mean_rp_volume = mean(rp_volume, na.rm = TRUE),
      sd_rp_volume = sd(rp_volume, na.rm = TRUE),
      .groups = "drop"
    ) %>%
    mutate(analysis = "all_case")
)
```
