# Supplementary material for: Robotic Surgical System Adoption and Radical Prostatectomy Volume in Japan: A Nationwide Hospital-Level Longitudinal Difference-in-Differences Study
Source: Int J Health Policy Manag. 2026 Aug 22;15:9571. doi: 10.34172/ijhpm.9571 (PMC13770223; doi:10.34172/ijhpm.9571)
Supplement: Supplementary file 2 [file ijhpm-15-9571-s002.pdf]

Article title: Robotic Surgical System Adoption and Radical Prostatectomy Volume in Japan: A Nationwide Hospital-Level Longitudinal Difference-in-Differences Study

Journal name: International Journal of Health Policy and Management (IJHPM)

Authors' information: Satoshi Funada[1]*, Jun Kamei[2], Yan Luo[3], Rei Goto[4,5]

[1]Health Technology Assessment Unit, Department of Preventive Medicine and Public Health, Keio University School of Medicine, Tokyo, Japan.

[2]Department of Urology, Graduate School of Medicine, The University of Tokyo, Tokyo, Japan.

[3]Department of Next-Generation Organ Transplantation, Graduate School of Medicine, The University of Tokyo, Tokyo, Japan.

[4]Graduate School of Business Administration, Keio University, Yokohama, Japan.

[5]Graduate School of Health Management, Keio University, Fujisawa, Japan.

***Correspondence to:** Satoshi Funada; Email: sfunada@keio.jp

Citation: Funada S, Kamei J, Luo Y, Goto R. Robotic surgical system adoption and radical prostatectomy volume in Japan: a nationwide hospital-level longitudinal difference-in-differences study. Int J Health Policy Manag. 2026;15:9571. doi:10.34172/ijhpm.9571

Supplementary file 2.

Table S1: Baseline Characteristics of Included Complete-Case Hospitals and Excluded Hospitals

Table S2: Trends in Radical Prostatectomy Volume by Robotic Surgical System Adoption Status from FY2016 to FY2023 (Complete-Case Analysis)

Table S3: Trends in Radical Prostatectomy Volume by Robotic Surgical System Adoption Status from FY2016 to FY2023 (All-Case Sensitivity Analysis)

Table S4: Estimated Average Treatment Effects on Radical Prostatectomy: All-Case Sensitivity Analysis

Table S5: Estimated Average Treatment Effects on Radical Prostatectomy: Stacked Difference-in-Differences Analysis

Figure S1: Trends in the Mean Annual Number of Radical Prostatectomies per Hospital by Robotic Surgical System Adoption Status, FY2016–FY2023: All-Case Sensitivity Analysis

Figure S2: Estimated Average Treatment Effects of Robotic Surgical System Adoption on Radical Prostatectomy: All-Case Sensitivity Analysis

Figure S3: Estimated Average Treatment Effects of Robotic Surgical System Adoption on Radical Prostatectomy: Stacked Difference-in-Differences Analysis

Figure S4: Comparison of Event-Study Estimates Between the Staggered Difference-in-Differences and Stacked Difference-in-Differences Analyses

Table S1. Baseline Characteristics of Included Complete-Case Hospitals and Excluded Hospitals

	**Included**			**Excluded**		
	**Previous adopters**	**New adopters**	**Non-adopters**	**Previous adopters**	**New adopters**	**Non-adopters**
**Variable**	**N = 189**	**N = 77**	**N = 22**	**N = 13**	**N = 185**	**N = 206**
Number of beds, mean (SD)	667 (245)	576 (184)	479 (199)	408 (331)	447 (179)	361 (137)
Number of physicians*, mean (SD)	246 (167)	175 (122)	117 (124)	128 (233)	109 (71)	69 (43)
Annual new inpatient cases, mean (SD)	18,362 (7,386)	16,546 (5,984)	11,945 (5,147)	8,188 (7,574)	11,611 (5,981)	8,529 (4,445)
Total number of surgeries, mean (SD)	604 (253)	519 (195)	413 (264)	292 (262)	368 (204)	267 (149)
Prefecture size, n (%)						
Large (≥3 million population)	109 (57.7%)	53 (68.8%)	8 (36.4%)	7 (53.8%)	108 (58.4%)	101 (49.0%)
Medium (1 to 3 million population)	62 (32.8%)	19 (24.7%)	13 (59.1%)	4 (30.8%)	62 (33.5%)	92 (44.7%)
Small (<1 million population)	18 (9.5%)	5 (6.5%)	1 (4.5%)	2 (15.4%)	15 (8.1%)	13 (6.3%)
Ownership, n (%)						
National government	53 (28.0%)	6 (7.8%)	6 (27.3%)	1 (7.7%)	10 (5.4%)	37 (18.0%)
Public organizations	67 (35.4%)	46 (59.7%)	8 (36.4%)	5 (38.5%)	94 (50.8%)	89 (43.2%)
Non-profit medical corporation	22 (11.6%)	4 (5.2%)	1 (4.5%)	5 (38.5%)	45 (24.3%)	44 (21.4%)
Private medical schools	27 (14.3%)	13 (16.9%)	5 (22.7%)	1 (7.7%)	8 (4.3%)	10 (4.9%)
Other	20 (10.6%)	8 (10.4%)	2 (9.1%)	1 (7.7%)	28 (15.1%)	26 (12.6%)

*Physician data were unavailable in FY2016 and were instead obtained from FY2017.

Abbreviation: FY, fiscal year; SD, standard deviation

Table S2. Trends in Radical Prostatectomy Volume by Robotic Surgical System Adoption Status from FY2016 to FY2023 (Complete-Case Analysis)

	**Previous adopters**	**New adopters**	**Non-adopters**	**Previous adopters**	**New adopters**	**Non-adopters**	**Previous adopters**	**New adopters**	**Non-adopters**	**Previous adopters**	**New adopters**	**Non-adopters**
	**N = 189**	**N = 77**	**N = 22**	**N = 189**	**N = 77**	**N = 22**	**N = 189**	**N = 77**	**N = 22**	**N = 189**	**N = 77**	**N = 22**
**(Adopted/Not yet)**		**(0/77)**			**(21/56)**			**(31/46)**			**(51/26)**	
		**2016**			**2017**			**2018**			**2019**	
Total	12,196	2,608	756	12,703	3,003	840	12,679	3,221	791	13,119	3,457	874
Mean (SD)	64.5 (34)	33.9 (18)	34.4 (34)	67.2 (33)	39.0 (22)	38.2 (33)	67.1 (32)	41.8 (25)	36.0 (31)	69.4 (34)	44.9 (27)	39.7 (33)
**(Adopted/Not yet)**		**(58/19)**			**(65/12)**			**(70/7)**			**(77/0)**	
		**2020**			**2021**			**2022**			**2023**	
Total	12,078	3,478	786	12,013	3,942	823	13,144	4,497	913	12,787	4,371	864
Mean (SD)	63.9 (33)	45.2 (25)	35.7 (24)	63.6 (34)	51.2 (28)	37.4 (26)	69.5 (37)	58.4 (30)	41.5 (29)	67.7 (37)	56.8 (27)	39.3 (26)

(Adopted/Not yet) indicates the number of hospitals that had already adopted robotic surgical systems and those that had not yet adopted within the new-adopter cohort in each fiscal year.
Abbreviation: FY, fiscal year; SD, standard deviation

Table S3. Trends in Radical Prostatectomy Volume by Robotic Surgical System Adoption Status from FY2016 to FY2023 (All-Case Sensitivity Analysis)

	Previous adopters	New adopters	Non-adopters	Previous adopters	New adopters	Non-adopters	Previous adopters	New adopters	Non-adopters	Previous adopters	New adopters	Non-adopters
		**2016**			**2017**			**2018**			**2019**	
	**N = 198**	**N = 135**	**N = 135**	**N = 201**	**N = 147**	**N = 130**	**N = 199**	**N = 158**	**N = 112**	**N = 197**	**N = 168**	**N = 109**
**(Adopted/Not yet)**		**(0/135)**			**(32/115)**			**(55/103)**			**(101/67)**	
Total	12,783	3,788	2,642	13,375	4,532	2,579	13,348	5,136	2,332	13,778	5,986	2,417
Mean (SD)	64.6 (35)	28.1 (18)	19.6 (17)	66.5 (34)	30.8 (21)	19.8 (17)	67.1 (33)	32.5 (24)	20.8 (17)	69.9 (36)	35.6 (23)	22.2 (18)
		**2020**			**2021**			**2022**			**2023**	
	**N = 198**	**N = 172**	**N = 94**	**N = 197**	**N = 202**	**N = 93**	**N = 198**	**N = 234**	**N = 98**	**N = 200**	**N = 258**	**N = 101**
**(Adopted/Not yet)**		**(130/42)**			**(162/40)**			**(214/20)**			**(258/0)**	
Total	12,695	6,610	2,083	12,440	8,421	2,077	13,651	10,684	2,443	13,437	11,418	2,659
Mean (SD)	64.1 (34)	38.4 (26)	22.2 (16)	63.1 (34)	41.7 (29)	22.3 (16)	68.9 (37)	45.7 (32)	24.9 (19)	67.2 (37)	44.3 (29)	26.3 (17)

(Adopted/Not yet) indicates the number of hospitals that had already adopted robotic surgical systems and those that had not yet adopted within the new-adopter cohort in each fiscal year.
Abbreviation: FY, fiscal year; SD, standard deviation

Table S4. Estimated Average Treatment Effects on Radical Prostatectomy: All-Case Sensitivity Analysis

	No. of hospitals	Average Treatment Effect	95% CI (Lower)	95% CI (Upper)
**Years since adoption**				
-6		1.00	-3.03	5.02
-5		-2.84	-5.76	0.07
-4		-1.07	-3.28	1.15
-3		1.75	-1.31	4.82
-2		-1.04	-3.36	1.28
-1		0.36	-2.22	2.95
0		7.84	4.98	10.70
1		12.13	8.35	15.91
2		15.58	10.86	20.30
3		14.48	9.05	19.90
4		14.03	8.29	19.77
5		11.73	4.23	19.22
6		9.07	0.05	18.09
**Groups by year of adoption**				
FY2017	32	11.00	4.30	17.69
FY2018	23	8.27	0.69	15.84
FY2019	46	16.49	7.41	25.56
FY2020	29	16.96	9.74	24.19
FY2021	32	16.06	0.20	31.91
FY2022	52	4.59	-1.11	10.29
FY2023	44	3.16	-2.73	9.05
**Overall average**		11.95	8.48	15.41

Event time was defined relative to the FY of robotic surgical system adoption (event time 0). Negative values indicate years before adoption and positive values indicate years after adoption. CI indicates confidence interval.

Abbreviation: CI, confidence interval; FY, fiscal year.

Table S5. Estimated Average Treatment Effects on Radical Prostatectomy: Stacked Difference-in-Differences Analysis

Years since adoption	Average Treatment Effect	95% CI (Lower)	95% CI (Upper)
0	3.66	-0.40	7.71
1	8.62	3.54	13.70
2	10.69	5.45	15.92
3	7.91	2.30	13.52
4	8.78	2.99	14.56
5	9.04	0.96	17.12
6	7.00	-2.56	16.57

Abbreviation: CI, confidence interval.

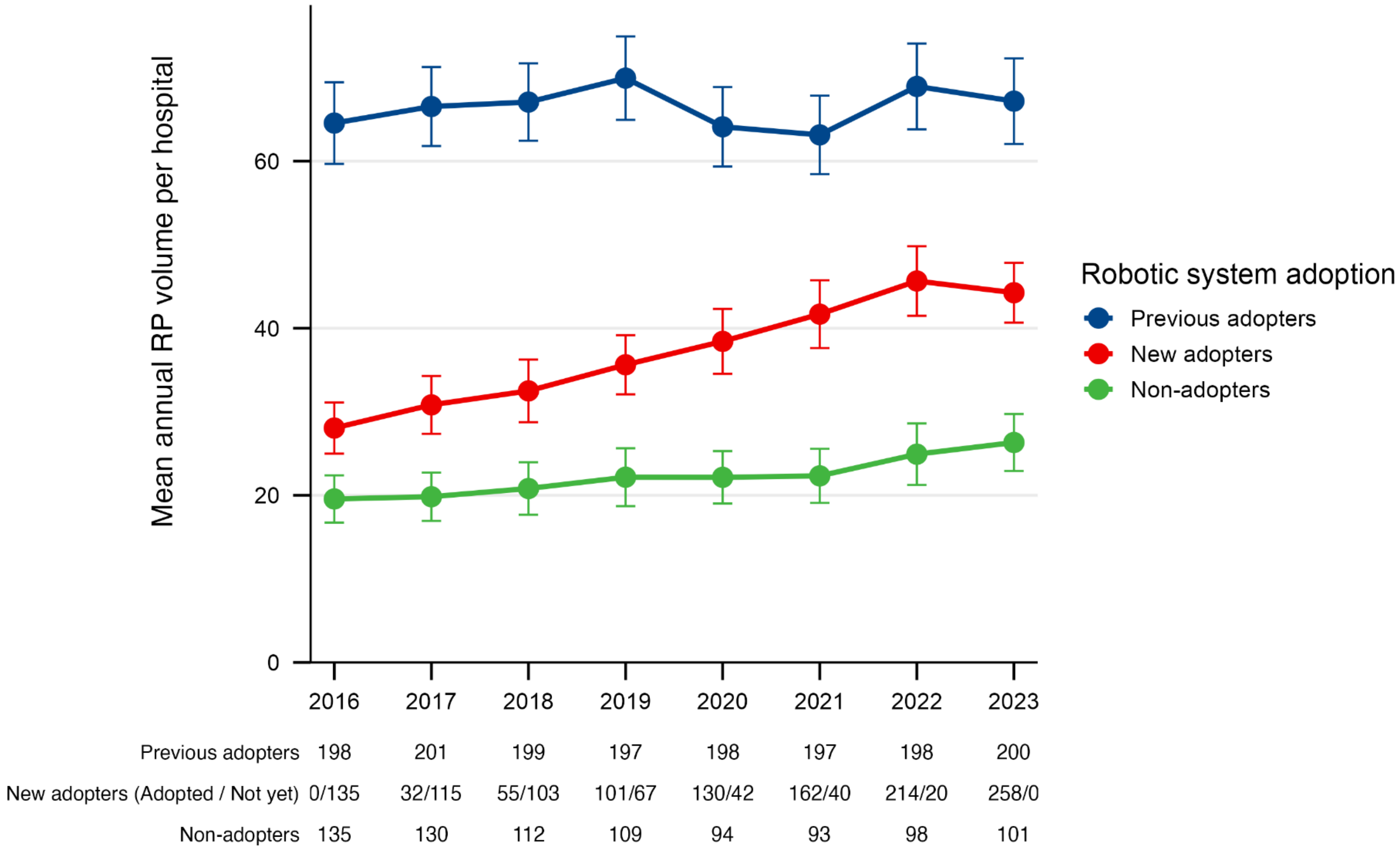

Figure S1. Trends in the Mean Annual Number of Radical Prostatectomies per Hospital by Robotic Surgical System Adoption Status, FY2016–FY2023: All-Case Sensitivity Analysis. Values represent mean annual RPs per hospital. Error bars indicate 95% confidence intervals. For new adopters, “Adopted/Not yet” indicates the number of hospitals that had already adopted robotic surgical systems and those that had not yet adopted in each fiscal year.

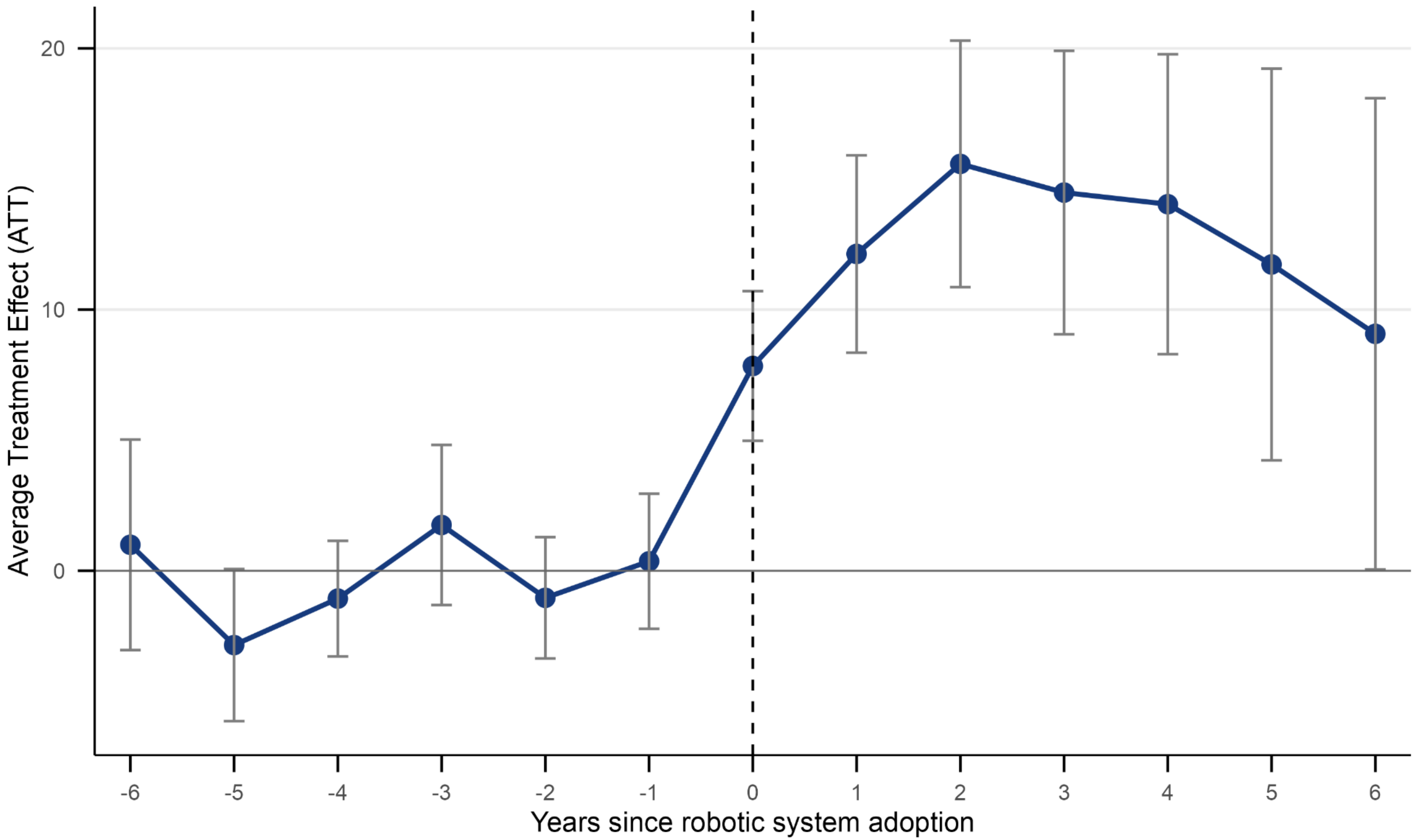

Figure S2. Estimated Average Treatment Effects of Robotic Surgical System Adoption on Radical Prostatectomy: All-Case Sensitivity Analysis. Event-study estimates using a staggered difference-in-differences approach. The dashed vertical line indicates the fiscal year of robotic surgical system adoption (event time 0). Negative event times represent years before adoption, and positive event times represent years after adoption. Error bars indicate 95% confidence intervals.

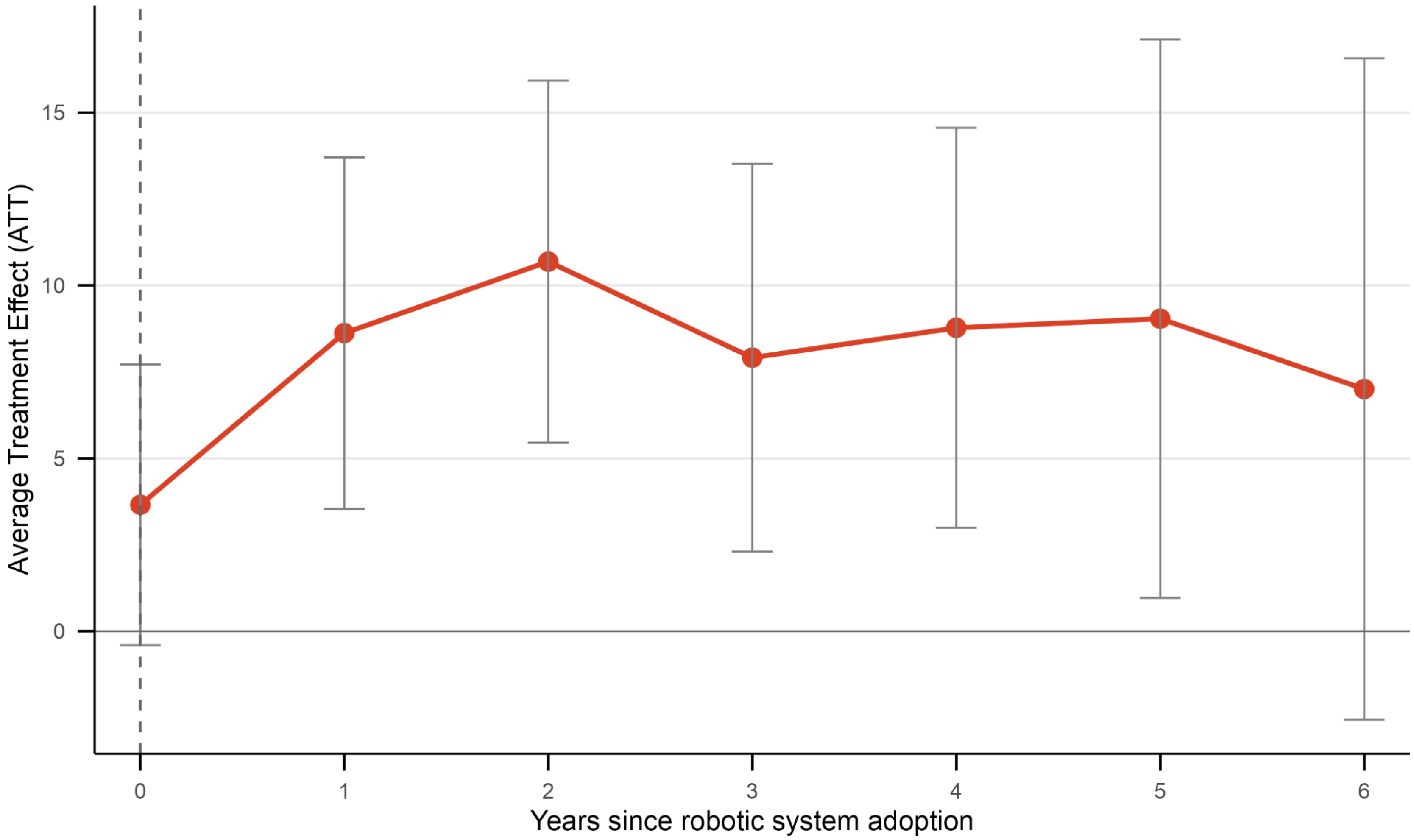

Figure S3. Estimated Average Treatment Effects of Robotic Surgical System Adoption on Radical Prostatectomy: Stacked Difference-in-Differences Analysis. The dashed vertical line indicates the fiscal year of robotic surgical system adoption (event time 0).

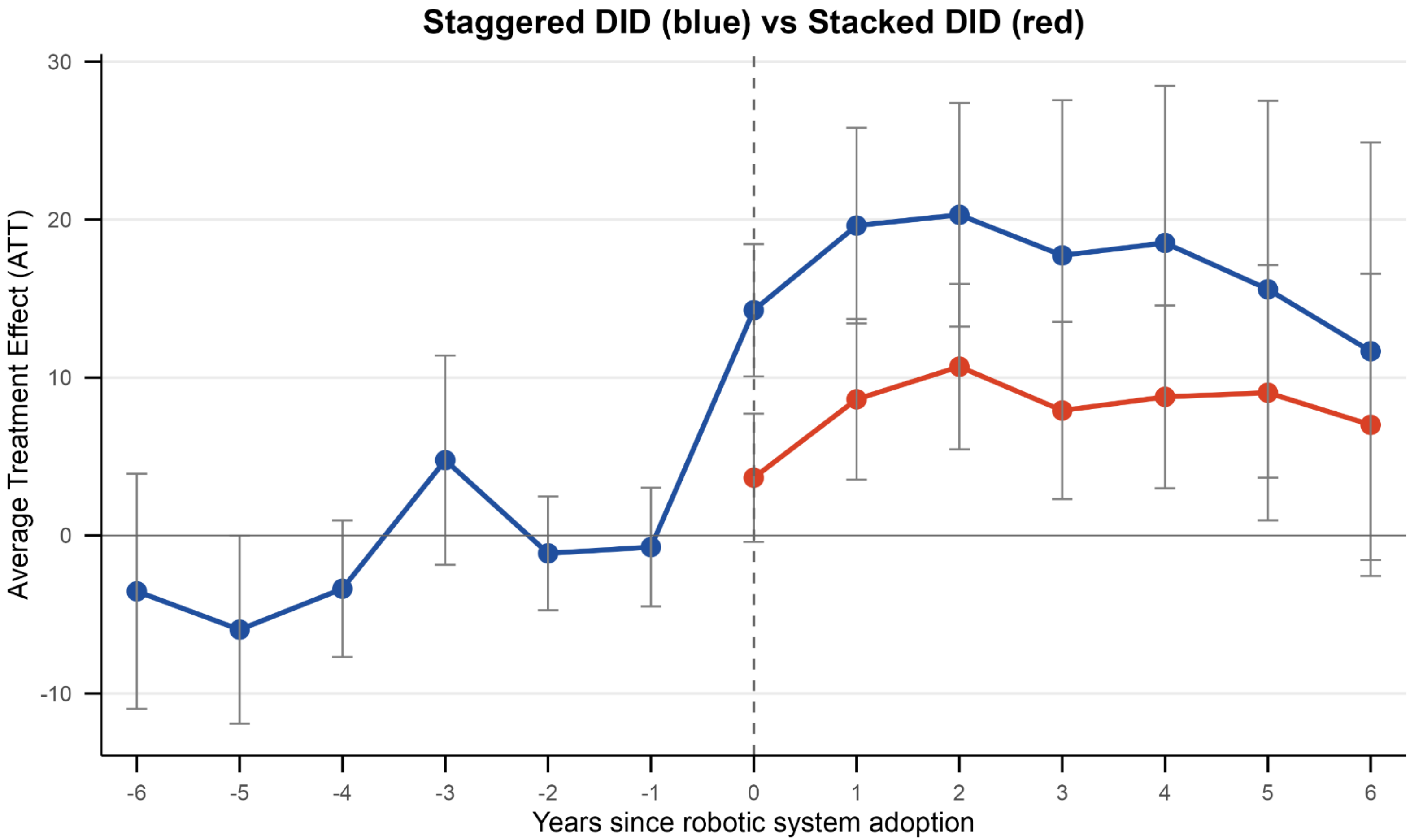

Figure S4. Comparison of Event-Study Estimates Between the Staggered Difference-in-Differences and Stacked Difference-in-Differences Analyses. The dashed vertical line indicates the fiscal year of robotic surgical system adoption (event time 0). Negative event times represent years before adoption, and positive event times represent years after adoption. Blue points and lines represent estimates from the staggered difference-in-differences analysis, whereas red points and lines represent estimates from the stacked difference-in-differences analysis. Error bars indicate 95% confidence intervals.
